# Associations Between Food Insecurity and Common Mental Health Problems Among Reproductive-Aged Women in Kabul-Afghanistan

**DOI:** 10.3389/fnut.2021.794607

**Published:** 2022-01-03

**Authors:** Fawzia Zahidi, Madiha Khalid, Pamela J. Surkan, Leila Azadbakht

**Affiliations:** ^1^Department of Community Nutrition, School of Nutritional Sciences and Dietetics, Tehran University of Medical Science (TUMS), Tehran, Iran; ^2^Toxicology and Disease Group, Pharmaceutical Sciences Research Center (PSRC), The Institute of Pharmaceutical Sciences (TIPS), Tehran University of Medical Sciences (TUMS), Tehran, Iran; ^3^Department of International Health, Bloomberg School of Public Health, Johns Hopkins University (JHU), Baltimore, MD, United States; ^4^Diabetes Research Center, Endocrinology and Metabolism Clinical Sciences Institute, Tehran University of Medical Sciences (TUMS), Tehran, Iran

**Keywords:** food insecurity, mental health problems, depression, anxiety, stress

## Abstract

**Background:** Food insecurity has been linked to poor health outcomes, however this relationship is poorly understood among women of reproductive age. Therefore, we investigated the relationship between food insecurity and common mental health problems (CMHPs) in this population of women in Kabul, Afghanistan.

**Method:** A cross-sectional study was conducted with 421 women of reproductive age from four health centers located in four randomly selected zones in the city of Kabul. We used the United State Department of Agriculture (USDA) food-insecurity questionnaire, multiple 24-h recall for dietary intake, the Depression, the Anxiety and Stress Scale (DASS-21) to assess major mental health problems, and the International Physical Activity Questionnaire (IPAQ) to assess physical activity.

**Result:** Food insecurity affected 69.6% of reproductive-aged women. In total, 44.9, 10.9, and 13.9% of food-insecure participants had food insecurity without hunger, food insecurity with hunger, and food insecurity with severe hunger, respectively. Depression, anxiety, and stress were prevalent among food-insecure participants at 89.4, 90.8, and 85.7%, respectively. Food insecurity was associated with depression (OR = 4.9, 95% CI: 2.7–8.9), anxiety (OR = 4.7, 95% CI: 2.5–8.8), and stress (OR = 3.8, 95% CI: 2.2–6.7). Women's household ownership, family size, and hypertension, on the other hand, were not associated with food insecurity.

**Conclusion:** This study found food insecurity was associated with CMHPs among a sample of reproductive-aged women in Kabul, Afghanistan. Further longitudinal studies are needed to confirm these findings.

## Introduction

The Food and Agriculture Organization (FAO) describes food insecurity (FI) as a condition in which people have limited access to sufficient nutritious and safe food to live a healthy and productive life (1). Food security, on the other hand, is defined as having enough, safe, and nutritious food to live a healthy and active life (1). Food and nutrition insecurity are important public health issues that affect millions of people globally (2). In 2018, 9.2% of people globally had severe food insecurity, with Sub-Sahara Africa and Asia having the highest rates of severe food-insecurity (3). Household food insecurity (HFI) has a negative impact on the nutritional status and health of vulnerable people, particularly women of reproductive age (4).

Recent studies have found that women who live in food insecure homes are more likely to have inadequate dietary intake (4), depression, and poor mental health (5). Common mental health problems (CMHPs) linked to FI include depression, anxiety, stress, and sleeping disorders (6–8). According to Laraia et al., food shortages and FI are associated with poor mental and physical health among women in the United States (6). A systematic review of FI and mental health among females in high-income countries found a relationship between depression and FI, as well as a relationship between the severity of HFI and chronic stress (9).

As a conflict afflicted country, Afghanistan's health statistics are among the poorest in the world (10, 11). According to research by the Global Network Against Food Crises, approximately two quarters of Afghans suffered from FI in 2018 (12). In a population-based study related to mental health and disability among participants over age 15 years in Afghanistan, depression, anxiety, and posttraumatic stress disorder (PTSD) were prevalent in 68, 72, and 42% of participants, respectively. These mental health problems were more common in women than in men (13). In Afghanistan, there is still a scarcity of data on the relationship between FI and mental health. Therefore, the aim of this study was to determine the association of FI with CMHPs among reproductive-aged women in Kabul, Afghanistan.

## Methods

Kabul has 22 municipalities with more than 160 health centers (14). This cross-sectional study was conducted in four municipalities, at four different health centers that were chosen using multistage random sampling. The aim that we include this four directions, was to include these four major ethnicities of Afghanistan in this present study; In the West mostly Hazara people are living, in the North mostly Tajik People, and in the East and South mostly Pashtun, Tajik and Uzbak people are living (15). Three comprehensive health centers (CHCs) were selected from the 15th (in the North), 13th (in the West), and 9th municipalities (in the East) (16). In the South, one district hospital (DH) was selected from the 16th municipality (16). From those health centers, a convenience sample of 421 reproductive-aged women were sampled by the following formula (17):


$$\begin{array}{l} \alpha =0.05\\ \text{Z}=95\%\;\left(1.96\right) \end{array}$$

*P* = 47 (*P* = 47%, the prevalence of overweight among wealthy reproductive-aged women) (18).


$$\begin{array}{l} q=\left(1-0.47\right)=\;\left(0.53\right)\\ n=\frac{{\left(Z_{1-\;\frac{\alpha }{2}}\right)}^{2}pq\;}{{\left(r\right)}^{2}}=\frac{{\left(1.96\right)}^{2}\;0.47\;x\;0.53\;}{{\left(0.05\right)}^{2}}\;=\;382.7\approx 383 \end{array}$$

For the study drop out or non-response coverage we add the 10% on our sample size which is calculated as $10\%\;of\;participant\;=\;\frac{10^{*}383}{100}\;=\;38$ person. Then we added 38 on to our total sample size, **38** **+** **383** **=** **421** participants. An equal number of routine visitor women (n = 105) were sampled from each of the four health centers.

### Assessment of Food Insecurity

In this study, we used the United States Department of Agriculture (USDA) 18-item questionnaire to assess FI (19, 20). This questionnaire reflects HFI in the last 12 months. In 1995, it was introduced as a valid questionnaire for epidemiologic studies and calculated based on the method of Bickel et al. (20).

### Assessment of Dietary Intake

All participants' dietary intakes were obtained using a 24-recall questionnaire on 3 days of the week (two during the week and one on the weekend) (21). Interviewers used a variety of tools to enhance participant reporting of serving sizes, including can sizes, a chunk of bread that fits in the palm of a hand, tablespoons, teaspoons, ladles, plates, bowls, glasses, and photographs of common household meals. We used the Automatic Multiple Pass Method (AMPM) to reduce bias in the 24-h recall questionnaire (22), which asks about (i) foods listed by participants that were consumed in the previous day, (ii) any forgotten foods, (iii) the time of day each food was consumed, (iv) specific details about foods (e.g., quantities consumed, and foods eaten between meals); and (v) whether anything was forgotten. Following that, portions were estimated based on household eating/cooking equipment and converted into grams from reported quantities, before being entered into Nutritionist 4 (NUT4) software for nutrient adequacy analysis.

### Assessment of Anthropometric Indices

Anthropometric indices, such as weight, height, and body mass index (BMI) were measured and computed for all participants. BMI was calculated by dividing weight (kg) by height^2^ (cm). A calibrated digital scale (SECA 831, Germany) was used for weight measurements. Adult BMI classifications according to the World Health Organization (WHO) are as follows: BMI <18.5 is considered low weight, BMI >18.5 <24.9 is considered normal weight, BMI >25 <29.9 is considered overweight, and BMI ≥30 is considered obese (23).

### Assessment of Common Mental Health Problems

The Depression, Anxiety and Stress Scale-21 Items (DASS-21) is a set of self-reported scales used to assess the CMHPs such as depression, anxiety, and stress. The DASS-21 scale has three sections, each of which comprises seven items (24). Dysphoria (a feeling of overall unhappiness with life), hopelessness, devaluation of one's life, self-deprecation, lack of interest, anhedonia (inability to experience pleasure), and inertia (a tendency to do nothing) are assessed on the depression component. Autonomic arousal, skeletal muscular responses, and subjective sensations of anxious affect are measured on the anxiety component. The stress component is sensitive to non-specific stimulant levels that have been present for a long time. CMHPs were categorized into three categories: normal, moderate, and severe.

### Assessment of Physical Activity

The International Physical Activity Questionnaire (IPAQ) was developed in the late 1990s to collect international comparable data on health-related physical activity. We used the long version of the IPAQ instrument (IPAQ-27 items) in this study (25).

### Statistical Analysis

The quantity of nutrients consumed by each participant was calculated using Nutritionist IV software. The data were analyzed using the Statistical Package for Social Science (SPSS Version 26) software. Histograms and the Kolmogorov-Smirnov tests were used to assess the normality of distributions of the variables. For general characteristics of individuals, the Chi-square test was performed, and one-way ANOVA was used to compare the means of categorical variables among FI categories. To assess the risk of CMHPs based on FI status, we fitted logistic regression models.

## Results

The data were collected from February to May 2021 and included an equal number of participants from each of the four health centers (*N* = 105 at each). Hazara, Tajik, Pashtun, and Uzbak ethnicities made up the majority of participants, accounting for 33.5, 32.8, 27.8, and 5.9%, respectively. The mean age and BMI of all participants were 31 ± 9 years and 23.3 ± 5.06 kg/m^2^, respectively. We also found the mean age 29.8 ± 9.1 years and mean BMI 24.2 ± 6.3 kg/m^2^ amongst FS participants, while among FI without hunger, FI with hunger and FI with severe hunger participants the mean age was 30.8 ± 8.9, 29.8 ± 9.3, and 35.3 ± 9.0 years and mean BMI was 23.0 ± 4.5, 23.1 ± 4.4, and 22.7 ± 3.5 kg/m^2^, respectively.

The average home size was 6 ± 3 people, with more than 91% of women having a family size of <10. The mean monthly income was US $241.4 ± 204.3, with 79.1% having a monthly income of < $300 and 14.3% having a monthly income of $500–1,200. In addition, the mean monthly income among FS participants $374 ± 275 were higher than food insecure participant's $183 ± 126. Meanwhile, the mean of monthly income was $194 ± 109, $168 ± 130, and $167 ± 160 among FI without hunger, FI with hunger, and FI with severe hunger.

Half of the women were illiterate, with just 14.8% having a high level of education (bachelor's or master's degree). We found that 30.4% (*n* = 129) of reproductive-aged women were food secure, whereas a large number of women (*n* = 293; 69.6%) were food insecure, in addition, more than three quarters of reproductive-aged women in West and South municipalities were suffering from FI. According to the food-insecurity categories, 44.9% (*n* = 189), 10.9% (*n* = 46), and 13.8% (*n* = 53) of participants, respectively, had food insecurity with hunger, food insecurity with mild hunger, and food insecurity with severe hunger. About four fifths of the women (81%) had low levels of physical activity, whereas 29% had at least a moderate level of physical activity.

In this study, food insecurity categories were significantly related to age, marital status, household size, and income (*P* < 0.05). Furthermore, ethnicity, education, and women's education level, as well as the occupation and education level of their husbands, were found to be associated with food insecurity (*P* = 0.001) (Table 1). BMI, hypertension, and physical activity, on the other hand, were not associated with food-insecurity categories. We found that the overall prevalence of depression, anxiety, and stress were 79, 81, and 74.6%, respectively, while the prevalence of these conditions among food insecure people were 89, 91, and 87%, respectively (Table 2). We found that common mental health problems (depression, anxiety, and stress) were significantly associated with food insecurity levels (*P* = 0.001). Severe depression, anxiety, and stress affected nearly 85, 88, and 84% of the food insecure population, respectively (Table 3). We found that FI was associated an increased risk of symptoms of depression (OR = 4.9; CI: 2.7–8.9), anxiety (OR = 4.7; CI: 2.5–8.8), and stress (OR = 3.8; CI: 2.2–6.7). Women's BMI >25 (OR = 1.3, CI: 0.7–2.4), household ownership (OR = 0.7, CI: 0.2–1.7), family size (OR = 1; CI: 0.6–1.9), and hypertension (OR = 0.6, CI: 0.2–1.9) were not associated with FI status (Table 4).

**Table 1:** Socio-demographic characteristic by different food-insecurity categories among reproductive-aged women in Kabul-Afghanistan.

**Background**	**Food-security status**		***p*-value**	**Food-insecurity categories**			*p*-value^*^
	**Food-secure *N* (%)**	**Food insecure *N* (%)**		**FI without hunger *N* (%)**	**FI with moderate hunger *N* (%)**	**FI with severe hunger *N* (%)**	
**Marital status**							
Single Married Widow	51 (47.7) 73 (24.9) 4 (19)	56 (52.3) 220 (75.1) 17 (81)	0.001	39 (36.4) 114 (49.1) 6 (28.6)	11 (10.3) 34 (11.6) 1 (4.8)	6 (5.6) 42 (14.3) 10 (47.6)	0.001
**Income** (US $)							
Less than 300$ 300–500$ 500–1,200$	68 (20.4) 31 (58.5) 29 (82.9)	265 (79.6) 22 (41.5) 6 (17.1)	0.001	167 (50.2) 19 (35.8) 3 (8.6)	42 (12.6) 2 (3.8) 2 (5.7)	56 (16.8) 1 (1.9) 1 (2.9)	0.001
**Household-size (number)**							
Less than 10 More than 10	121 (30.9) 7 (23.3)	270 (69.1) 23 (76.7)	0.04	179 (45.8) 10 (33.3)	43 (11.0) 3 (10.0)	48 (12.8) 10 (33.3)	0.015
**Ethnicity**							
Pashtun Tajik Hazara Uzbak	32 (27.4) 58 (42) 25 (17.7) 13 (52)	85 (72.8) 80 (58) 116 (82.3) 12 (48.0)	0.001	71 (60.7) 58 (62) 54 (38.3) 6 (24)	8 (6.8) 11 (80) 24 (17) 3 (12)	6 (5.1) 11 (8) 38 (19.4) 3 (3.4)	0.001
**Municipalities**							
North West East South	52 (49.1) 12 (11.4) 38 (36.2) 26 (24.8)	54 (50.9) 93 (88.6) 67 (63.8) 79 (75.2)	0.0001	30 (28.0) 41 (39.0) 53 (50.5 65 (61.9)	14 (13.2) 21 (20.0) 5 (4.8) 6 (5.7)	10 (9.4) 31 (29.5) 9 (8.6) 8 (7.6)	0.0001
**Education level**							
Illiterate Primary Secondary High school Bachelor Master	39 (17.6) 10 (25.6) 8 (24.2) 25 (37.9) 38 (73.1) 8 (80)	182 (82.4) 29 (74.4) 25 (75.8) 41 (62.1) 14 (26.9)	0.001	110 (49.8) 16 (41) 18 (54.4) 34 (51.5) 9 (17.3) 2 (20)	31 (14) 7 (17.9) 3 (9.1) 3 (4.5) 2 (3.8) 0 (0)	41 (18.6) 6 (15.4) 4 (12.1) 4 (6.1) 3 (5.8) 0 (0)	0.001
**Husband's education level**							
Illiterate Primary Secondary High school Bachelor Master	14 (10.6) 6 (18.2) 4 (16) 17 (45.9) 30 (49.2) 6 (60.0)	121 (89.6) 27 (81.8) 21 (84) 20 (54.0) 31 (50.8) 4 (40.0)	0.001	71 (52.6) 18 (54.5) 16 (64) 12 (32.4) 24 (39.3) 4 (40.0)	22 (16.3) 5 (15.2) 2 (8) 3 (8.1) 4 (6.6) 0 (0)	28 (20.7) 4 (12.1) 3 (12) 5 (13.5) 3 (4.9) 0 (0)	0.001
**Occupation**							
Jobless Worker Employee Scientific member	68 (21.6) 18 (30) 37 (90.2) 4(50)	242 (78.1) 42 (70) 4 (9.8) 4 (50)	0.001	157 (50.6) 25 (41.7) 3 (7.7) 4 (50)	36 (11.6) 9 (15) 1 (2.4) 0 (0)	49 (15.8) 8 (13.3) 0 (0) 0 (0)	0.001
**Husband's occupation**							
Jobless Worker Farmer Employee Scientific member	0 (0) 34 (18.3) 1 (16.7) 30 (42.9) 7 (70)	18 (100) 152 (81.7) 5 (83.3) 40 (57.1) 3 (30.0)	0.001	11 (61.1) 97 (52.2) 1 (16.7) 30 (42.9) 3 (30)	4 (22.2) 23 (12.4) 1 (16.7) 7 (10) 0 (0)	3(16.7) 32 (17.2) 3 (50) 3 (4.3) 0 (0)	0.001
**Hypertension illness**	10 (7.8)	23 (7.8)	0.581	14 (7.4)	4 (8.7)	5 (8.6)	0.986
**Physical activity**							
Low Moderate Intensive	108 (31.7) 20 (26.0) 0 (0)	233 (68.3) 57 (74.0) 3 (100)	0.001	152 (44.6) 35 (45.5) 2 (66.7)	36 (10.6) 10 (13.0) 0 (0)	45 (13.2) 12 (15.6) 1 (33.3)	0.719

* *P-values are based on the chi-square test*.

*FI, food-insecurity*.

**Table 2:** Mental health status among reproductive-aged women in Kabul, Afghanistan.

**Mental health**	**Food secure *N* (%)**	**Food insecure *N* (%)**	*P*-value^*^	**FI without hunger *N* (%)**	**FI with hunger *N* (%)**	**FI with severe hunger *N* (%)**	***P*-value**
Depression	70 (54.7)	262 (89.4)	0.001	161 (85.2)	43 (93.5)	58 (100)	0.001
Anxiety	76 (59.4)	266 (90.8)	0.001	164 (86.8)	44 (95.7)	58 (100)	0.001
Stress	63 (49.2)	251 (85.7)	0.001	153 (81.0)	40 (87.0)	58 (100)	0.001

* *P-values are based on Chi-square tests*.

**Table 3:** Mental health status in different food-insecurity categories among reproductive-aged women in Kabul, Afghanistan.

**Major mental health problems**	**Level**	**Food-insecurity status**		***P*-value**	**Food-insecurity categories**			*P*-value*
		**Food- Security *N* (%)**	**Food-insecurity *N* (%)**		**FI without hunger *N* (%)**	**FI with mild hunger *N* (%)**	**FI with severe hunger *N* (%)**	
Depression	Normal	58 (45.3)	31 (10.6)	0.001	28 (14.8)	3 (6.5)	0 (0)	0.001
	Moderate	8 (6.3)	13 (4.4)		10 (5.3)	3 (6.5)	0 (0)	
	Severe	62 (48.4)	249 (85.0)		151 (79.9)	40 (87.7)	58 (100)	
Anxiety	Normal	52 (40.6)	27 (9.2)	0.001	25 (13.2)	2 (4.3)	0 (0)	0.001
	Moderate	8 (6.3)	9 (3.1)		7 (3.7)	2 (4.3)	0 (0)	
	Severe	68 (53.1)	257 (87.7)		157 (83.1)	42 (91.3)	58 (100)	
Stress	Normal	65 (50.8)	42 (14.3)	0.001	36 (19.0)	6 (13.0)	0 (0)	0.001
	Moderate	3 (2.3)	4 (1.4)		3 (1.6)	0 (0)	1 (1.7)	
	Severe	60 (46.9)	247 (84.3)		150 (79.4)	40 (87.0)	57 (98.3)	

* *P-values are based on Chi-square tests*.

*FI, Food-insecurity*.

**Table 4:** Odds ratios (ORs) of common mental health problems according to food-insecurity status among reproductive-aged women in Kabul-Afghanistan.

Variables^**^	**Food-insecure vs. food-secure unadjusted OR (95% CI)**			**Food-insecure vs. food-secure adjusted OR (95% CI)**		
	**OR**	**CI**	***P*-value**	**OR**	**CI**	*P*-value^*^
Depression	7.0	4.2–11.6	0.001	4.9	2.7–8.9	0.001
Anxiety	6.7	3.9–11.4	0.001	4.7	2.5–8.8	0.001
Stress	6.1	3.8–9.9	0.001	3.8	2.2–6.7	0.001
BMI >25	1.4	0.8–2.2	0.138	1.3	0.7–2.4	0.255
Hypertension	1.0	0.4–2.1	0.990	0.7	0.2–1.7	0.471
Household ownership	1.5	0.9–2.2	0.056	1.0	0.6–1.7	0.753
Family size >10 members	1.4	0.5–3.3	0.449	0.6	0.2–1.9	0.455

* *P-values are based on binary logistic regression*.

** *All the variables were adjusted for BMI, income, marital status, education level, occupation, ethnicity, family size, financial level, and house ownership*.

## Discussion

In this study, we found that about 70% of reproductive-aged women were food insecure, nearly half were food insecure, and 11, 14, and 11%, respectively, were affected by FI without hunger, FI with hunger, and FI with severe hunger. Findings show that the prevalence of FI in our study is nearly 40% higher than the National Risk and Vulnerability Assessment (NRVA) in 2011/2012, reporting food and nutrition insecurity to be about 30% (7.6 millions) among Afghans generally (26). The high level of FI status among reproductive-aged women in this study may have been elevated due to the civil war and the COVID-19 pandemic. Depressive symptoms were present in 79% of reproductive-aged women, whereas symptoms of anxiety and stress were present in 81 and 75%, respectively. In contrast to our findings, a national survey of anxiety disorders and major depressive episodes conducted in 2021 found that the prevalence of general anxiety disorder and major depressive episode were significantly lower, at 11.7 and 2.7%, respectively (27). However, such higher prevalence may be expected since our study used symptom measures (interview) rather than evaluation of clinical diagnoses (observation, psychological tests, neurological tests, and interviews) (28). Based on our study, 89, 90, and 85% of FI participants had symptoms of depression, anxiety, and stress, respectively. Further, FI was associated with having these symptoms.

Consistent with our results, a growing body of evidence suggests that FI is linked to common mental health problems (29–31). A systematic review of females in developed countries found a strong relationship between FI and depression and stress (9). Lachance et al. found a positive relationship between FI and mental health problems in a quantitative community-based study of Canadians. They also concluded that FI and mental health problems, particularly depression, had a bidirectional relationship, with poor mental health often causing people to make poor food choices (32). In contrast to our study, Chung et al. investigated the relationship between household FI and adverse mental health problems in Korean adults and found that FI was associated with stress, anxiety and depression (29). Similarly, Scanlon et al. investigated depression and social vulnerability in African-American men and found that FI did not enhance the likelihood of depression (33). Moreover, we found a high prevalence of depression, anxiety and stress among FS participants, it may be because of gender-based violence, civil war and insecure situation in Afghanistan especially in Kabul (34).

We also found that sociodemographic factors such as household income and education level were associated with FI status. In comparison to their husbands, most participating women were uneducated. This may be due to a variety of factors, including civil wars, poverty, minority status, early marriage and pregnancy, and gender-based violence, which impede women and girls from fully exercising their education (35). Omidvar et al. observed that household income, socioeconomic status, and education level among Afghan refugees in Iran were associated with FI (36). Similarly, in a study on food insecurity and its determinants in Nigeria, Amaza et al. found that household income, education level, and gender were the most important indicators of FI status (37).

We also found that marital status and household size were associated with food insecurity. Married women with children are more prone to utilize risky coping techniques like restricting food intake to ensure that their children and other household members are well-fed (38). A study from a South African Township indicated that marital status, household income, and household size were significantly associated with FI (39). However, in another study of South African households, marital status and household size were negatively associated with FI (40). Among socio-demographic factors we found age, ethnicity and house ownership to also be associated with FI. These findings were similar to those of Fernandes et al.'s cohort study, which indicated that 23% of older adults lived in a food-insecure households. The odds of FI was higher for participants in the 70–74 year old age category (41). In a study of FI, depression, and race among university students, Reeder et al. reported that African-American students had 3.5 times higher odds of FI than Caucasian students (42). Similarly, a US study of diet quality and FI among people of various races found that FI was most prevalent among non-Hispanic white individuals and Asians.

We found that FI is associated with depression, anxiety and stress. More research is needed to confirm these findings and to examine if the same patterns hold true for different populations (e.g. in different regions in Afghanistan, age groups, etc.). Furthermore, additional development activities, such as economic and education programs, could be beneficial for Afghan women to improve their home food security. Limitations of this study include that it was a cross-sectional study that only included reproductive-aged women, with no other age groups or males. Our study also had several strengths. We used the USDA long version scale for FI measurements, the DASS-21 for CMHPs measurements, and it was the first study to examine the relationship between FI and CMHPs in reproductive-aged women in Kabul, Afghanistan.

## Conclusion

A considerable number of women of reproductive age in our study, over two-thirds, were food insecure. Notably, almost 25% reported food insecurity with hunger or food insecurity with severe hunger. Common mental health problems were extremely prevalent among food-insecure individuals, with 90% of this subgroup displaying symptoms for depression and anxiety and 86% reporting stress. FI was associated with about a five-fold risk of depression and anxiety and about a four-fold risk of stress. Women's household ownership, family size, and hypertension were not associated with food insecurity status. Given the strong association between FI and CMHPs among reproductive-aged women and high prevalence of FI in Kabul policies should prioritize access to food among these women. Furthermore, due to the high prevalence of CMHPs a psychoanalytic intervention should be done for reproductive-aged women.

## Data Availability Statement

The raw data supporting the conclusions of this article will be made available by the authors, without undue reservation.

## Ethics Statement

The studies involving human participants were reviewed and approved by Tehran University of Mediccal Sciences Ethical approval ID (IR.TUMS.MEDICINE.REC.1399.656). Written informed consent to participate in this study was provided by the participants' legal guardian/next of kin.

## Author Contributions

LA conceptualized this study. FZ and MK designed the study. FZ performed statistical analysis. The study was finalized by LA and PS who contributed to the writing and interpretation of the results. All authors read and approved the final manuscript.

## Funding

This research was a MSc thesis project, which was funded by Tehran University of Medical Sciences (TUMS Grant No. 99-2-163-49080).

## Conflict of Interest

The authors declare that the research was conducted in the absence of any commercial or financial relationships that could be construed as a potential conflict of interest.

## Publisher's Note

All claims expressed in this article are solely those of the authors and do not necessarily represent those of their affiliated organizations, or those of the publisher, the editors and the reviewers. Any product that may be evaluated in this article, or claim that may be made by its manufacturer, is not guaranteed or endorsed by the publisher.
